# Optimization of the Pixel Design for Large Gamma Cameras Based on Silicon Photomultipliers

**DOI:** 10.3390/s24186052

**Published:** 2024-09-19

**Authors:** Carolin Wunderlich, Riccardo Paoletti, Daniel Guberman

**Affiliations:** 1 Istituto Nazionale di Fisica Nucleare (INFN), Sezione di Pisa, 56126 Pisa, Italy; carolin.cw.wunderlich@web.de (C.W.); riccardo.paoletti@pi.infn.it (R.P.); 2 Dipartimento di Scienze Fisiche, della Terra e dell’Ambiente, Università di Siena, 53100 Siena, Italy; 3 Departament de Física Quàntica i Astrofísica (FQA), Universitat de Barcelona (UB), 08028 Barcelona, Spain; 4 Institut de Ciències del Cosmos (ICCUB), Universitat de Barcelona (UB), 08028 Barcelona, Spain

**Keywords:** SPECT, silicon photomultiplier (SiPM), gamma camera, large-area SiPM

## Abstract

Most single-photon emission computed tomography (SPECT) scanners employ a gamma camera with a large scintillator crystal and 50–100 large photomultiplier tubes (PMTs). In the past, we proposed that the weight, size and cost of a scanner could be reduced by replacing the PMTs with large-area silicon photomultiplier (SiPM) pixels in which commercial SiPMs are summed to reduce the number of readout channels. We studied the feasibility of that solution with a small homemade camera, but the question on how it could be implemented in a large camera remained open. In this work, we try to answer this question by performing Geant4 simulations of a full-body SPECT camera. We studied how the pixel size, shape and noise could affect its energy and spatial resolution. Our results suggest that it would be possible to obtain an intrinsic spatial resolution of a few mm FWHM and an energy resolution at 140 keV close to 10%, even if using pixels more than 20 times larger than standard commercial SiPMs of 6 × 6 ${\mathrm{mm}}^{2}$. We have also found that if SiPMs are distributed following a honeycomb structure, the spatial resolution is significantly better than if using square pixels distributed in a square grid.

## 1. Introduction

Gamma cameras based on large scintillator crystals [1] and large (4–8 cm diameter) photomultipliers (PMTs) are still widely used in single-photon emission computed tomography (SPECT). In a typical SPECT gamma camera, the angular acceptance of the incoming gamma rays is limited using a lead collimator. Since the scintillator and PMTs must be surrounded with a thick layer of lead to block radiation arriving from other directions, a SPECT camera can weight a few hundred kilograms. If the weight of a SPECT camera could be reduced, it should be possible to soften the requirements needed to build a SPECT system, allowing to build more compact scanners that could fit in smaller rooms and be safer to operate.

As pointed out in [2], more compact and lighter SPECT systems could be achieved if replacing the PMTs, which occupy about 50% of the camera volume, with compact detectors like silicon photomultipliers (SiPMs). SiPMs have other advantages, like providing a higher photodetection efficiency (PDE) or being insensitive to magnetic fields. A SiPM-based gamma camera appears also as a low-cost alternative to those based on cadmium–zinc–telluride (CZT) detectors [3,4,5]. CZT-based SPECT cameras should outperform scintillator-based cameras, at least in terms of energy resolution, but they are also much more expensive, which could be particularly relevant when building large cameras.

Several works have studied the possibility of using SiPMs in gamma cameras. The INSERT project proposed to introduce compact small gamma cameras based on SiPMs as *inserts* that could be compatible with MRI [6]. In [7], the authors developed a hand-held SiPM-based gamma camera for intraoperative imaging. A side-by-side phoswich detector combined with a SiPM array was proposed for SPECT imaging, using LYSO/GAGG as scintillators instead of NaI or CsI [8]. In [9], a simulation study of the optimal design of a Philips DPC3200 SiPM photosensor-based thin monolithic scintillator detector for SPECT applications was published. It described a four-side buttable detector stack which allowed tiling to generate large surface area MRI-compatible radiation detectors, ideal for SPECT applications. The use of digital SiPMs for small SPECT cameras was also studied in [10]. A novel benchtop SiPM-based preclinical SPECT system optimized for in vivo mouse imaging was characterized in [11].

All these SiPM-based solutions for SPECT applications were designed targeting small cameras. Scaling this solution to a large camera is hard due to the lack of commercial large-area SiPMs, which are hardly found in sizes larger than 6 × 6 ${\mathrm{mm}}^{2}$. Filling a full-body SPECT camera with sensors of this size would *a priori* require to process thousands of readout channels, which would dramatically increase the cost and complexity of the system. The limitation to build larger SiPMs arises from the fact that their capacitance increases dramatically with size, which comes with a huge degradation of the signal-to-noise ratio (SNR) and the time resolution.

In [2], we proposed an alternative solution to build large SPECT cameras based on SiPMs with a reasonable amount of readout channels. This solution employed large-area SiPM pixels (LASiPs), which were built by summing the currents of a few SiPMs into a single output. With this approach it is possible to achieve larger SiPM pixels while keeping the capacitance at a reasonable level. To study the feasibility of using this solution in SPECT, we constrcuted a proof-of-concept micro-camera that consisted of a 40 × 40 × 8 ${\mathrm{mm}}^{3}$ NaI(Tl) crystal coupled to four LASiPs, each of them built from the sum of eight SiPMs of 6 × 6 ${\mathrm{mm}}^{2}$. We were able to reconstruct simple images of radioactive sources with an intrinsic spatial resolution of ∼2 mm and measured an energy resolution of ∼11.6% at 140 keV. We also simulated the micro-camera with Geant4 and were able to optimize its parameters and validate it by achieving a very good agreement with our experimental results.

In [12], we discussed for the first time the possibility of extending the simulations of the micro-camera to a much larger camera, similar in size to that of a standard full-body SPECT scanner, in order to study the feasibility of using LASiPs in full-body SPECT. We also introduced a reconstruction algorithm based on iterative methods as an improvement with respect to the centroid algorithm we had used in [2]. In [13] we showed our first preliminary results of those simulations, in which we compared the spatial resolution obtained with LASiPs of different sizes and where we also simulated different SiPM noise levels. Those results also suggested that when the SiPMs that build a LASiP were grouped following non-square geometries the spatial resolution could improve.

This work represents a detailed study that completes what we started in [12] and continued in [13]. With the simulation that had been previously validated with a experimental micro-camera and that was extended to match in size a full-body SPECT camera, we aimed at providing tools that could be useful towards an optimization of a large SiPM-based gamma camera. Here, we provide for the first time a deep study of the dependence of the energy and spatial resolution with the pixel size, shape and noise.

## 2. Materials and Methods

The main goal of this work was to study the feasibility of having a full-body SPECT camera equipped with LASiPs by evaluating the impact that the pixel size and shape would have on the energy and spatial resolution of the camera. To do so, we extended the Geant4 simulations of the LASiP-SPECT micro camera. that were performed and validated in [2] to a much larger camera, similar in size to that of standard clinical SPECT scanners. In this section, we describe these simulations and the charge and image reconstruction methods we applied. Other aspects that were not part of this study but that could be of primordial importance towards a large SiPM-based SPECT camera like power consumption or the necessity of a cooling system are briefly discussed in Section 4.

### 2.1. The LASiP Concept

In a LASiP, the analog currents of several SiPMs are summed into a single output. This way, the pixel size can be increased by a factor that is equal to the number of summed SiPMs while keeping the capacitance at a reasonable level, as it was shown and discussed in [2,14,15,16,17]. The SiPM signals are amplified, filtered and summed using custom-design circuits that are typically based on operational or common-base transistor amplifiers. This sum can be performed with discrete components or using an ASIC like the MUSIC (eight-channel Multiple Use IC for SiPM anode readout [17]) as we did in [2]. Using an ASIC offers several advantages, including compactness and ease of reproduction on a large scale, which are particularly important when building (several) large cameras.

### 2.2. Simulations of a Full-Body Spect Camera

In [2], we simulated a small SPECT micro-camera of 40×40×9 ${\mathrm{mm}}^{3}$, which we validated with experimental measurements. For this article we extended those simulations to build a full-body gamma camera equipped with a large NaI(Tl) crystal of 500 × 400 × 9 ${\mathrm{mm}}^{3}$ and a light yield of 38 photons/keV and SiPMs of 6 × 6 ${\mathrm{mm}}^{2}$ with a peak PDE of 50% and a crosstalk probability of 25%. The crystal was surrounded by a MgO reflector. A 1 mm fused-silica glass window was set between the crystal and the SiPMs. We used the *RoughTeflon LUT Davis* model [18] to describe the interaction of optical photons in the crystal–MgO reflector interface, since, in the simulations of the micro-camera, we found that it was the one in better agreement with our experimental data. We set the scintillator intrinsic resolution to achieve an energy resolution of ∼8% at 140 keV in the absence of SiPM noise. We simulated different radioactive sources of 140 keV: point-like sources, linear sources (capillaries of negligible diameter) and a 2-D source inspired by a Derenzo phantom. This source consisted of circles of increasing diameter *d* (1, 2, 3, 4, 6 and 8 mm) with a distance of 2d between neighboring circles.

**Figure 1:**
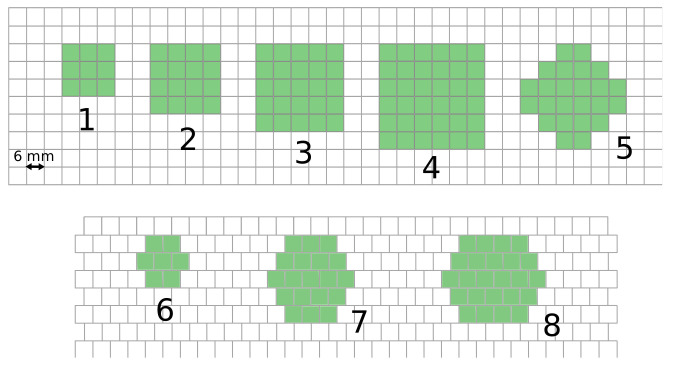
Scheme showing the different LASiP configurations (1–8).

Since we were only interested at the intrinsic resolution of the system, we did not simulate a collimator. Instead, all the 140 keV gamma rays were directed normally towards the NaI(Tl) crystal in all the simulated sources. To reduce computing time, the simulations were limited to a region of ∼225 × 225 mm around the center of the camera, where 1296 SiPMs were placed. We set a gap of 0.25 mm between neighboring SiPMs, which could be arranged following a square grid or a honeycomb geometry (see Figure 1). For each event, we recorded the number of photons collected by each SiPM. Then, we artificially added noise to the SiPM signals to account for the finite resolution of the system and to include the effect of optical crosstalk and especially dark counts, since in [2] we have shown that they were the main source of noise affecting the performance. We did not include afterpulsing, which we had shown to be negligible. Noise was added to the simulations following the steps described in Section 2.8 of [2]. This procedure includes a model that was optimized with experimental data and describes the degradation of the SiPM SNR as the LASiP size increases. We set an integration time of 600 ns, which was enough to collect more than 90 % of the scintillation photons while limiting the amount of integrated dark counts. For all our simulations, we assumed a rather high optical crosstalk probability of 25%. We simulated three different levels of dark count rate (DCR):

- ‘Cooled-SiPM’ noise: DCR = 0.015 MHz/mm^2^, representing standard SiPMs cooled-down to ∼4 °C;
- ‘Room-SiPM’ noise: DCR = 0.050 MHz/mm^2^, representing standard SiPMs at room temperature;
- ‘Hot-SiPM’ noise: DCR = 0.150 MHz/mm^2^, representing either standard SiPMs at high temperatures or rather old SiPMs at room temperature.

The SiPMs were arranged in two ways, following a square and a honeycomb geometry (see Figure 1). The SiPMs could then be summed and grouped to build LASiPs of different size and shape. All the LASiPs we built are listed in Table 1 and illustrated in Figure 1. LASiPs 1–4 and LASiPs 6–8 follow the ‘natural’ square/honeycomb geometry of the simulated SiPMs. In the case of LASiP 5, the SiPMs were placed in the camera following a square geometry, but were grouped in a way we called ‘flower’ geometry.

### 2.3. Charge Reconstruction

For each event, we registered the output charge $q_{i}$ of each pixel. The pixel output charge is the result of the contributions from the scintillation photons collected by that pixel and from the noise that was generated in it, as explained in Section 2.2. We then calculated the total charge *Q* collected by the *M* pixels with the highest individual output charge as $Q=\sum_{i}^{M}q_{i}$. We only kept those events with a total charge within $Q_{140}\pm 15\%$, where $Q_{140}$ was the mean charge associated to the 140 keV photopeak.

### 2.4. Image Reconstruction

We reconstructed the x,y coordinates of the scintillation events using the statistical method based on the Maximum-Likelihood Estimation (MLE) that we described in [12] and [13]. This method was inspired by the algorithms developed by [19,20,21]. It offers several advantages compared with traditional centroid algorithms (like the one we used in [2]): it typically provides a better spatial resolution, smaller distortions, a larger useful FOV and a better filtering of noise events (see for instance [22,23]). On the other hand, statistical reconstruction techniques are longer and demand more computing power.

#### 2.4.1. Light Response Function

The statistical reconstruction technique we employ requires a detailed knowledge of the spatial response of the detector. The key is to obtain the Light Response Function (LRF): the signal recorded by each pixel as a function of the position of the scintillation event. To do so we, simulated a γ-ray beam normally incident to the detector plane, which was placed in *N* calibration positions ($x_{cal},y_{cal}$). If $q_{ij}$ is the charge collected by the pixel *i* in a scintillation event generated when the beam was placed at ($x_{cal,j}$, $y_{cal,j}$), the total charge $Q_{j}$ collected by all *M* pixels would be:

(1) $$Q_{j}=\sum_{i}^{M}q_{ij}$$

For each ($x_{cal,j},y_{cal,j}$), we calculated the normalized charge distribution $q_{ij}/Q_{j}$ collected by the pixel *i* in each scintillation event and filled a 2D histogram. This histogram was interpolated and fitted with a generalized bell-shaped membership function [24]:

(2) $$\begin{array}{c} f\left(x,y,a,b,c,d,e\right)=\frac{a}{1+{\sqrt{{\left(\frac{x-d}{b}\right)}^{2}+{\left(\frac{x-e}{b}\right)}^{2}}}^{2c}} \end{array}$$

where a,b,c,d,e are free parameters of the fit. The resulting fit function was an estimate of the LRF ${\hat{f}}_{i}\left(x,y\right)$ of the pixel *i*. For more details, we refer the reader to [13].

In the simulations performed in this work, we built a square grid of N=21×21 calibration points. The center of the grid was placed at the center of the camera and had a lattice spacing of 6.5 mm.

#### 2.4.2. Maximum-Likelihood Estimation

The position of a scintillation event (x,y) is reconstructed by finding the set of coordinates ($\hat{x}$, $\hat{y}$) that maximize the likelihood *L* in Equation (3). When doing so, it is assumed that the probability for a pixel *i* to collect a charge *q* in a scintillator camera can be described by a Poisson distribution [23]. Details of the derivation of Equation (3) can be found in [13].

(3) $$\ln\left(L\left(\hat{x},\hat{y}\right)\right)=-\sum_{i}^{M}\left(q_{i}\cdot \ln\left(f_{i}\left(\hat{x},\hat{y}\right)\cdot \frac{\sum_{i}^{N}q_{i}}{\sum_{i}^{M}f_{i}\left(\hat{x},\hat{y}\right)}\right)-f_{i}\left(\hat{x},\hat{y}\right)\cdot \frac{\sum_{i}^{M}q_{i}}{\sum_{i}^{M}f_{i}\left(\hat{x},\hat{y}\right)}\right)$$

The maximization of Equation (3) was realized in the C++-based language root, using the Minuit2Minimizer class. As inputs we had the charge $q_{i}$ given by each pixel with coordinates $\left(x_{i},y_{i}\right)$ and their estimated LRF.

As as first estimation of (x,y), we used the central coordinates $(x_{max},y_{max)}$ of the pixel which detected the maximum charge. We constrained (x,y) to be within a certain diameter $r_{rec}$ from $(x_{max},y_{max)}$. The number of pixels used to reconstruct an event $M_{rec}$ was also optimized in order to maximize the SNR. The optimal values of $r_{rec}$ and $M_{rec}$ can in principle depend on the pixel size, the position of the source with respect to the pixels and the DCR level. We found that the impact of $r_{rec}$ in the accuracy of the reconstruction was negligible and that a minimization radius of ∼40 mm was good enough. We also found that $M_{rec}=21$ (the pixel with the maximum charge and its 20 nearest neighbours) provided the best result in terms of spatial resolution and reconstruction speed. Even in the absence of noise, we found that choosing larger values of $M_{rec}$ did not provide a significant improvement of the spatial resolution. Note that the number of pixels used to reconstruct the position does not need to be the same than the one used to reconstruct the energy.

#### 2.4.3. Spatial Linearity and Uniformity Corrections

Two corrections were applied to the images reconstructed following the method described in Section 2.4.1. As in [2], we applied a spatial linearity correction and an uniformity correction.

The spatial linearity correction was based on [7]. To do so we used the 2D calibration grid of point-like sources that we employed to estimate the LRF. Knowing the LRF, we reconstructed the position of all those point sources following the steps described in Section 2.4.1. For each point source *j*, we compared the reconstructed position $\left(x_{rec,j},y_{rec,j}\right)$ with its known real position $\left(x_{cal,j},y_{cal,j}\right)$. With this, we could build a 2D correction map that could be used to correct, by interpolation, the reconstructed images (see [2] for the details).

**Figure 2:**
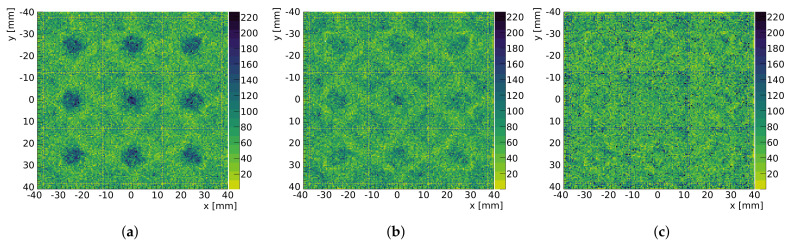
A simulated FFI using LASiP 2 without noise: (**a**) raw reconstruction, (**b**) after spatial linearity correction and (**c**) after uniformity correction. The blue dashed lines show the LASiP edges.

To build the uniformity correction map, we simulated a flat-field image (FFI). We reconstructed it, applied linearity correction, normalized it and finally inverted it. All reconstructed images were multiplied by this map (also here see [2] for more details). Figure 2 shows an example of a simulated FFI reconstructed with LASiP 2 pixels and without noise at three different stages: the raw reconstruction obtained after minimizing Equation (3) for each event (Figure 2a), after linearity correction (Figure 2b) and after uniformity correction (Figure 2c).

### 2.5. Energy Resolution

To calculate the energy resolution, we obtained the charge spectrum of the simulated images and identify the 140 keV photopeak. The energy resolution was defined as the ratio between the FWHM of that peak to its position (in units of energy). Both the peak position and its width were obtained by applying a Gaussian fit to the photopeak.

For the different noise levels and LASiP configurations, we sought the number of pixels that should be used to reconstruct the charge to optimize the energy resolution.

### 2.6. Intrinsic Spatial Resolution

To obtain the intrinsic spatial resolution, we simulated a 1-D linear (capillary) source, oriented in the *y* axis, located in different *x* positions. Since the response could have a dependence on the (x,y) position of the source, we took, for a single capillary located at $x_{i}$, several projections at different *y* positions. Each projection was modeled using a Cubic Spline interpolation performed in root using the TSpline3 class, with which we could extract the FWHM. The use of this method was a conservative approach from our side. We also tested the more conventional approach of fitting the projections with a Gaussian. But, we found that sometimes the projections did not fit very well to a Gaussian and that in those cases we were underestimating the actual FWHM of the distributions.

With all the FWHM values obtained (one per projection), we built a new distribution. The mean of that distribution was what we defined as the spatial resolution for a linear 1-D source located at $x_{i}$. The error of the spatial resolution was the standard deviation of that distribution. We compared the spatial resolution we obtained with all the LASiP configurations described in Table 1 and for all the noise levels simulated.

## 3. Results

### 3.1. Energy Resolution

**Figure 3:**
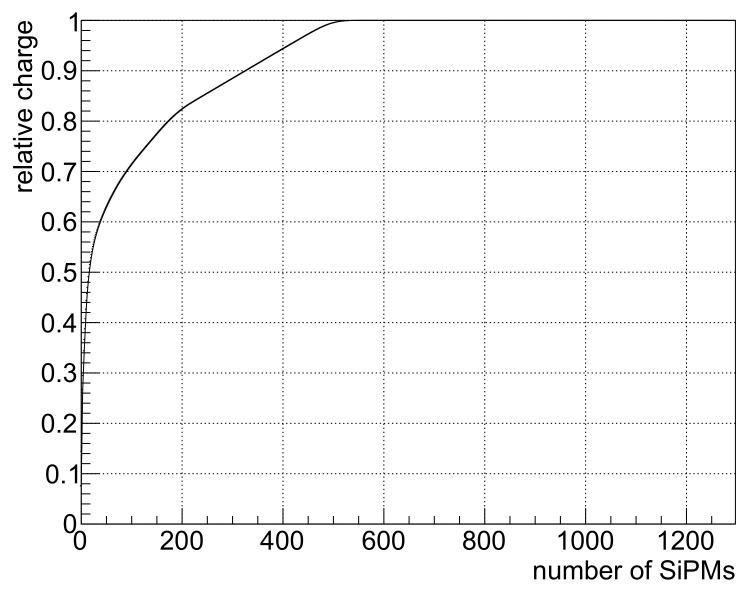
Collected charge (relative to the total number of collected photons) as a function of the number of SiPMs used to collect the charge.

**Figure 4:**
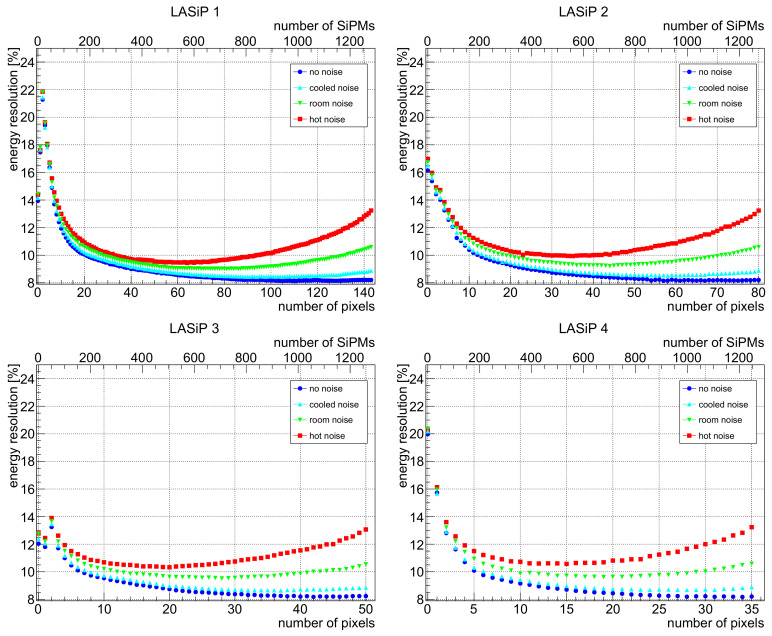
Energy resolution as a function of the number of LASiPs used to collect the charge (LASiP 1–4) under different SiPM noise levels. The top *x* axis shows the total number of SiPMs employed.

Figure 3 shows the total collected charge, in the absence of noise, as a function of the number of SiPMs used to collect it. It shows that in a typical scintillation event, most of the charge would be concentrated in a few tens of SiPMs and that ∼500 SiPMs would be enough to collect more than 99% of it. This is true while the SiPMs are read out individually and one is capable to sort them by the intensity of their signal. When SiPMs are grouped in LASiPs, this number increases a little bit, as can be inferred from Figure 4. This Figure shows the energy resolution we obtained as a function of the number of pixels used to collect the charge, using LASiP 1–4. The secondary *x* axis shows the equivalent number of SiPMs employed to collect the charge. The results are shown for all noise levels (including no noise at all). As expected, when there is no noise, the energy resolution improves as the number of SiPMs employed increases due to a more efficient light collection. For all LASiP configurations, the energy resolution reaches a plateau at ∼800–1000 SiPMs. The addition of noise has a significant impact in the energy resolution. When the DCR increases, the optimal number of LASiPs that should be used to optimize the energy resolution shifts towards lower values. This is expected, since at some point there will be pixels in which the number of integrated dark counts becomes comparable or larger than the number of collected photons. In the worst scenario (‘hot noise’), the optimal number of SiPMs employed would be between 500 and 600, which corresponds to ∼60 LASiP 1 or ∼15 LASiP 4 pixels. With a standard DCR of ∼0.05 kHz/mm^2^, the optimal energy resolution is obtained when ∼700 SiPMs are employed (∼80 LASiP 1 or ∼20 LASiP 4 pixels). In this case, the energy resolution goes above 9% in all pixels, which is more than 10% higher than in the absence of noise. Reducing the DCR by cooling down the SiPM temperature to a few degrees could help improving the energy resolution. Even in the absence of dark counts, there would still be a minor degradation of the energy resolution due to optical crosstalk. This is why the ‘cooled nooise’ curve is always higher than the ‘no noise’ one, even for low number of pixels.

Table 2 shows the energy resolution we obtained with LASiP 1–5, at all noise levels, using the optimal number of pixels for each configuration. In the absence of noise or when the noise level is low, the pixel size has almost no impact on the energy resolution. However, the degradation of the energy resolution with noise is more significant in larger pixels. With a DCR of 50 kHz/mm^2^, which is standard in commercial SiPMs operated at room temperature, the energy resolution obtained with LASiP 4 is more than 5% larger than the one obtained with LASiP 1. With higher noise levels, this difference is higher. The reason is that when using smaller pixels, it is easier to discard those pixels that are dominated by noise. LASiP 5 provides essentially the same energy resolution at all noise levels than LASiP 3, which is very close in size to LASiP 5 but has a different geometry. This would suggest, as it was expected, that the geometry of the pixels has no impact on the energy resolution of the camera.

### 3.2. Intrinsic Spatial Resolution

In this section we evaluate the impact that pixel size, shape and noise have in the spatial resolution.

#### 3.2.1. Impact of the Pixel Size

**Figure 5:**
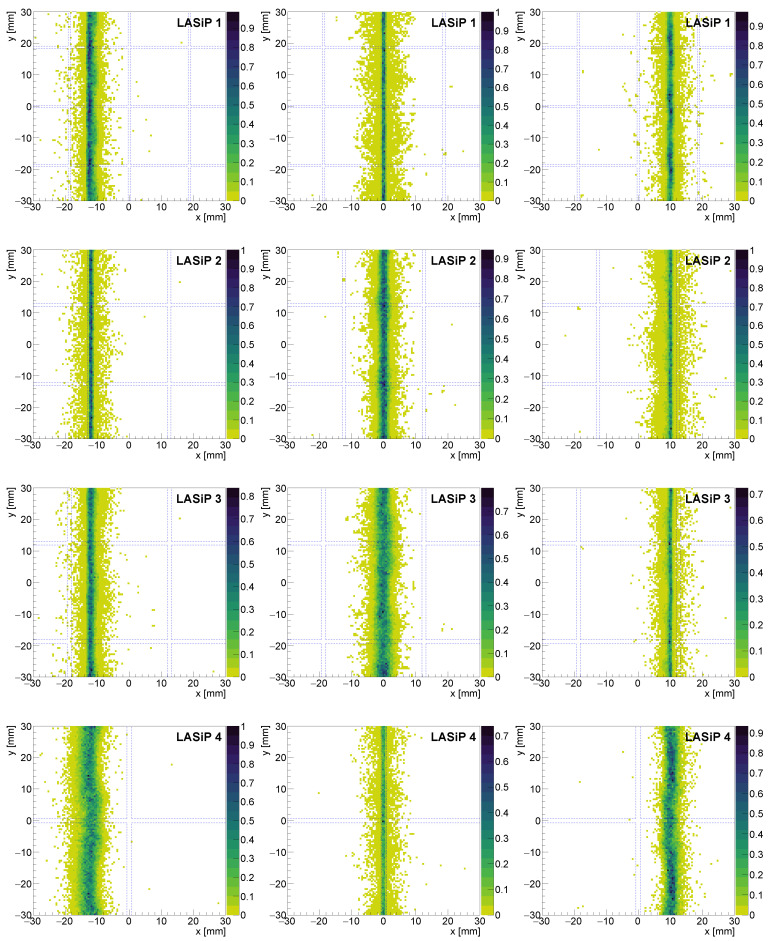
Reconstructed image of a capillary source at x=−12,0,10 mm for LASiP 1–4 at room-SiPM noise. The blue dashed lines mark the borders of the LASiPs.

To study the impact of the pixel size in the spatial resolution we compared the images obtained with all the pixels with square geometry (LASiP 1–4). Figure 5 shows the reconstructed images of capillary sources at different *x* positions (x=−12,0,10 mm) at room-SiPM noise. It can be qualitatively observed that the width of the reconstructed capillary increases with the pixel size. Additionally, when comparing the images reconstructed with the same LASiP, a dependence of the capillary width on its position in the *y* axis can also be appreciated. As will be discussed shortly afterwards, the spatial resolution seems to be worse when the capillary passes close to a pixel center.

**Figure 6:**
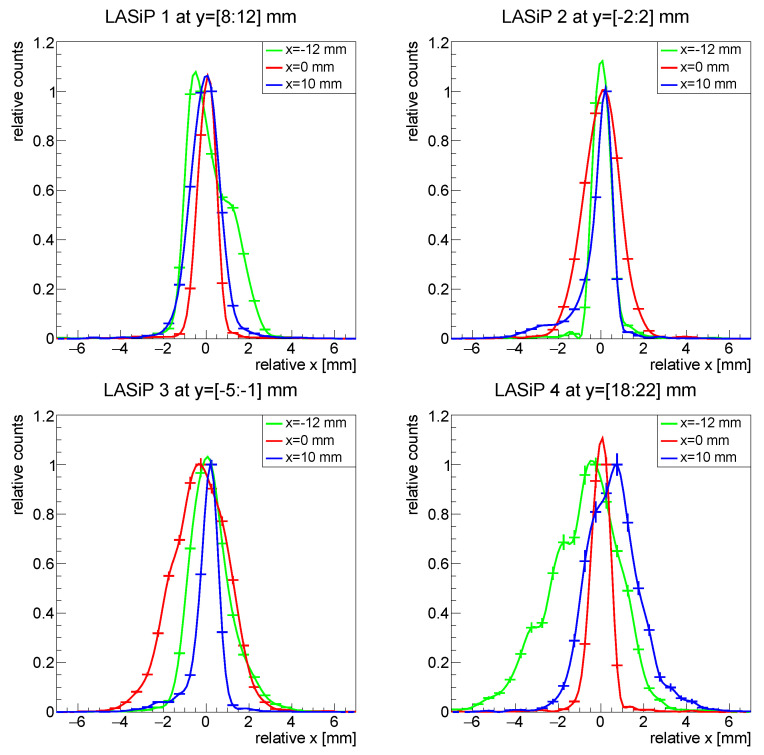
Normalized projections in the *x* axis of the reconstructed images of Figure 5. The projections were performed in a narrow region around the center of the pixel.

Figure 6 shows the projections in the *x* axis of all the images of Figure 5. To facilitate the comparison, all projections were shifted to be centered at x=0. To perform these projections, we selected only events with reconstructed *y* values within 2–3 mm from the $y_{j}$ coordinate of one of the pixels (see title of each panel in Figure 6). This was a conservative choice since, as can be inferred from Figure 5 and Figure 6 and as is discussed shortly after, the spatial resolution is better when the capillary appears close to a pixel edge than when it passes close to its center.

**Figure 7:**
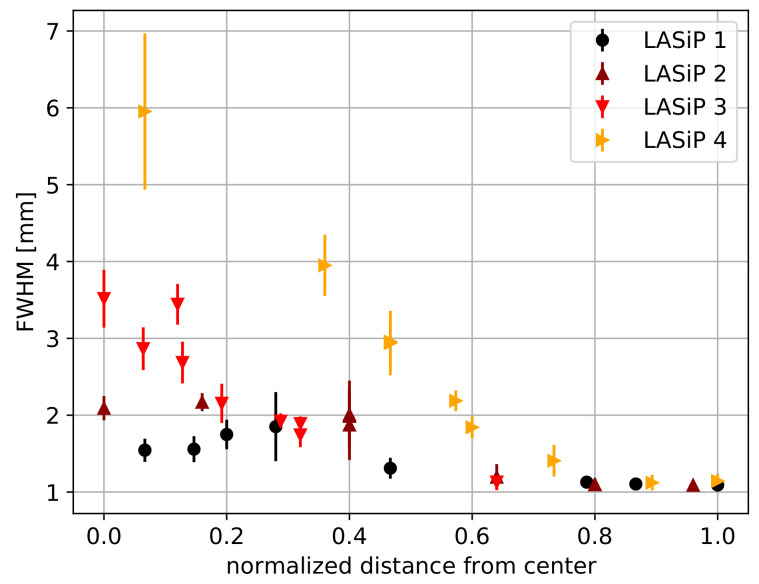
Mean FWHM of the reconstructed capillary as a function of the normalized distance to the pixel center $\hat{d}$ ($\hat{d}=0$ describes a capillary passing by the center of a pixel, $\hat{d}=1$ describes a capillary at the edge of a pixel).

Figure 7 compares the average FWHM of the capillaries reconstructed with LASiPs 1–4 as a function of the normalized distance to the center of the pixel $\hat{d}$. $\hat{d}$ is given in units of the pixel size. This way, $\hat{d}=0$ describes a capillary passing by the center of a pixel, while $\hat{d}=1$ describes a capillary at the edge, equidistant from two pixels. As expected, smaller pixels provide a better intrinsic spatial resolution, which can be below 2 mm for LASiP 1. Still, for LASiP 3, which is almost 3 times larger than LASiP 1 and 25 times larger than a commercial SiPM of 6×6 ${\mathrm{mm}}^{2}$, the intrinsic spatial resolution would be below 3 mm over almost all the area of the camera. The intrinsic spatial resolution is significantly worse for LASiP 4, although still acceptable if we compare it with standard SPECT scanners based on PMTs.

The dependence of the spatial resolution on the position of the source in the camera is related to the nature of the reconstruction mechanism. The achievable intrinsic spatial resolution is a result of the positional information provided to the reconstruction algorithm. When charge is shared among several pixels, the reconstruction tends to be more precise. If an event is recorded close to the pixel center, a single pixel will detect most of the photons (especially when the pixels are large). The reconstruction is less precise in this case because the relative contribution from the other pixels to the reconstructed position is sub-dominant. Part of these effects is compensated by the correction algorithms described in Section 2.4.3, but some artifacts may still remain, especially in the case of large LASiPs. Note that the dependence of the intrinsic spatial resolution with the position of the source is particularly noticeable in Figure 5 because we simulated an ideal 1-D linear source. Real capillary sources or masks made of high-density materials that are employed during the experimental characterization of gamma cameras have typical thickness of a few mm, and hence this spatial dependence would be smeared out.

The spatial resolution obtained with all LASiP configurations in the absence of noise and with room-SiPM is summarized in Table 3. The values of the spatial resolution given there are conservative because they were obtained for capillaries located close to the pixel center. By looking at LASiPs 1–4 (which have the same pixel shape), it can be seen that the spatial resolution gets worse as the pixel size increases. The spatial resolution is better in smaller pixels because it is easier to exclude those with a worse SNR, but mainly because they provide more information of the light distribution in a single scintillation event. This allows building more precise LRFs and a better estimation of the reconstructed position.

**Figure 8:**
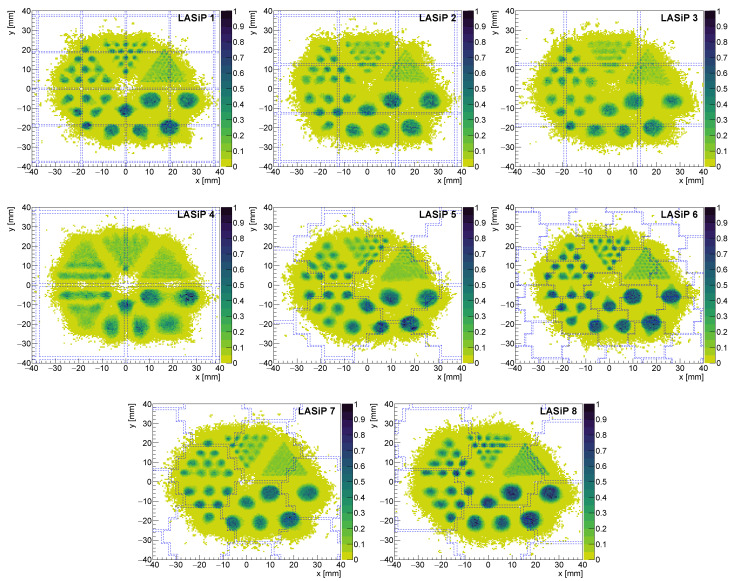
Reconstructed image of a simile-Derenzo source (the diameter of the circles are 1, 2, 3, 4, 6 and 8 mm) at room-SiPM noise. The dashed lines mark the edges of the LASiPs.

A more realistic source than our 1-D capillary is the simile-Derenzo phantom we also simulated. The reconstructed images of this phantom under room noise can be seen in Figure 8. The degradation of the spatial resolution with the pixel size can also be observed in the first four panels, which show the images reconstructed with LASiP 1–4. While with the largest pixel, LASiP 4, only those circles with diameter ≥ 6 mm can be resolved, with LASiP 1 it is possible to clearly resolve the circles with diameter ≥ 2 mm.

#### 3.2.2. Impact of the Pixel Geometry

**Figure 9:**
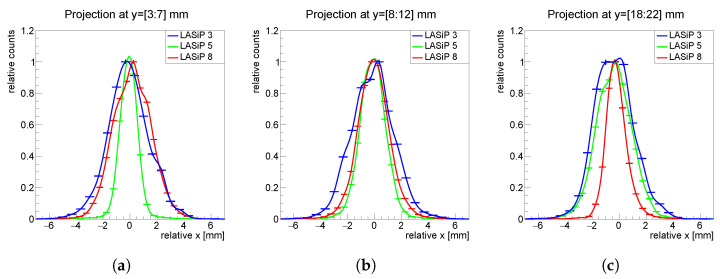
Normalized projections in the *x* axis of the reconstructed images of capillaries passing through the center of the LASiP 3, LASiP 5 and LASiP 8 pixels. The projections were centered at different *y* values: (**a**) y=5 mm, (**b**) y=10 mm, (**c**) y=20 mm.

The last four panels of Figure 8 show the reconstruction of the simile-Derenzo source with LASiPs 5–8, which are those that do not follow a square geometry. A degradation of the spatial resolution with the pixel size can also be seen when comparing LASiPs 6–8, although this degradation does not seem to be as strong as it is with LASiPs 1–4. The spatial resolution obtained with all 8 LASiP configuration is summarized in Table 3. Note that even if the pixel area of LASiPs 5 and 8 is very similar to the one of LASiP 3, the spatial resolution is significantly worse in the latter one. This effect can be seen in Figure 9, where we compare the projections in the *x* axis of a capillary passing through the pixel center in LASiPs 3, 5 and 8, measured at different *y* values. As discussed in Section 3.2.1, the spatial resolution improves with the distance to the closest pixel center. This explains why the width of the reconstructed projection is better for LASiP 5 in Figure 9a, but it is better for LASiP 8 in Figure 9c. However, the spatial resolution is systematically worse in LASiP 3. This would suggest that the honeycomb geometry should be preferable, at least in terms of spatial resolution. We attribute it to the fact that in a honeycomb geometry, a pixel has six nearest neighbors: overall, more pixels would give a significant contribution to the position reconstruction. LASiP 5, due to its ‘unnatural’ geometry, represents a special case. On average, it provides a better spatial resolution than LASiP 3, but its spatial dependence is larger (which explains why the relative errors of the calculated spatial resolution are also larger). This effect has been studied in detail in [13].

**Figure 10:**
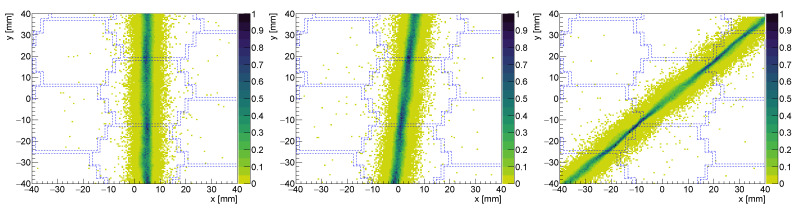
Reconstructed images of a 1-D capillary source using LASiP 8 at room-SiPM noise. The dashed lines mark the edges of the LASiPs.

To test the robustness of the reconstruction algorithm, we also simulated capillaries with different orientations. As an example, Figure 10 shows some images reconstructed with LASiP 8 under room-SiPM noise. Beyond the broadening of the capillary when it passes close to the pixel center, we do not observe any artifact in the reconstructed images.

#### 3.2.3. Impact of the Pixel Noise

**Figure 11:**
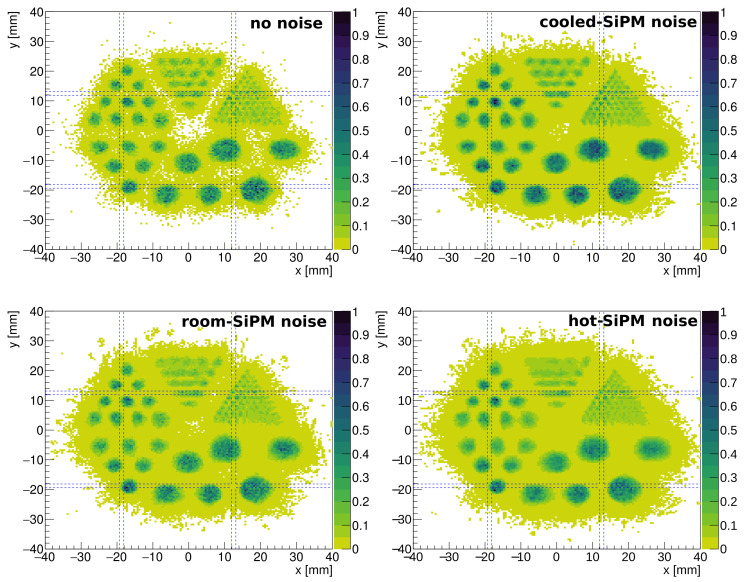
Reconstructed image of the simile-Derenzo source at different noise levels using LASiP 3. The dashed lines mark the edges of the LASiPs.

Figure 11 shows the reconstructed image of the simile-Derenzo source with LASiP 3 at different noise levels. There is a small degradation of the spatial resolution when going from *cooled* to *room* SiPM noise, which can be observed in the 2 mm circles. The degradation is naturally larger for *hot* SiPM noise.

Table 4 shows the calculated mean intrinsic spatial resolution for LASiPs 1–5 at all noise levels. There is a degradation of the spatial resolution due to SiPM noise, which is particularly relevant for large LASiPs (LASiPs 3–5). Dark counts are on average distributed equally among all SiPMs. As a result, when the image reconstruction is performed with smaller LASiPs, there are more pixels with a higher SNR (ratio of collected photons to integrated dark counts). In this case, the main impact that dark counts have is a sort of ‘blurring’ of the images which somehow softens the spatial dependence of the spatial resolution. This is why the spatial resolution is slightly degraded, but the relative error appears to be smaller. When using larger LASiPs, most of the collected photons are distributed in a few pixels. Those with fewer photons that still contribute to the image reconstruction will have a significantly worse SNR when dark counts are included. As a result, the spatial resolution becomes significantly worse with noise in these cases.

## 4. Discussion

In this article, we studied, through simulations, how the approach that we discussed in [2] of replacing the PMTs of traditional SPECT cameras with LASiPs could be extended to a full-body camera of 500×400×9 ${\mathrm{mm}}^{3}$. In particular, we focused on the impact that the pixel size, shape and noise could have in the spatial and energy resolution of the camera. The goal was to provide some tools that could be useful for someone who might consider designing a large gamma camera based on SiPMs. The values of energy and spatial resolution that we reported are useful to have an idea of the performance that could be achieved but were mostly useful to compare the impact of the different LASiP configurations we proposed. These numbers are also supported by a robust simulation that had previously been validated with experimental data. In [2], we were able to fine-tune parameters like the SiPM PDE and noise, the scintillation intrinsic resolution and the spatial distribution of the scintillation photons in order to reproduce with reasonable accuracy what we measured in the laboratory with our prototype camera. We should still remark that there are many effects that we did not simulate. For instance, for simplicity, we assumed that all SiPMs had the same gain. This choice was supported by [25], where the authors showed that eventual differences in the gain could be compensated by their reconstruction algorithm (which is similar to the one we used in this work). Additionally, even if we started from simulations that had been carefully optimized and validated with experimental data, the results presented in this work have not been contrasted with new experiments (it would require building a full-body LASiP-based SPECT camera). Pixels summing 14 SiPMs of 6×6 ${\mathrm{mm}}^{2}$ SiPMs have been built in [16] and were compatible with the strict timing and electronic noise requirements of Cherenkov telescopes for gamma-ray astrophysics. Since in SPECT those requirements would be softer, it is reasonable to expect that it would be feasible to employ LASiPs summing ∼20 SiPMs. It should be noted that the model we used to describe the degradation of the SNR of the LASiPs with the pixel was validated with LASiP summing signals from N=1 to 8 SiPMs and that we assumed that it was still valid for larger values of *N*. If a larger LASiP was built, the accuracy of this assumption should be tested. As a result, any comparison with a real SPECT camera should be made with caution. Still, even if the numbers we give should be considered just as a reference, they suggest that reasonable performance could be achieved using LASiPs, even at room temperature.

A standard clinical full-body SPECT camera based on PMTs provides a typical energy resolution of ∼10% at 140 keV [26]. The energy resolution depends on the characteristics of the scintillator, the photodetectors, the readout electronics and how efficiently they are combined. Strictly from the photosensor side, which is where this paper has put its focus, the higher the PDE and the lower the noise, the better the energy resolution. Since SiPMs offer a much better PDE (a typical value for commercial SiPMs and PMTs are ∼50% and ∼35% at ∼420 nm, respectively), they have the potential to provide a better energy resolution. However, in the system we simulated we found that when SiPMs are operated at room temperature dark counts can degrade the energy resolution by up to ∼15%, even after optimizing the number of pixels used to collect the charge. As a result, we expect the energy resolution of a full-body SPECT camera equipped with LASiPs operated at room temperature to be comparable to that of a camera based on PMTs. As we have shown, cooling the SiPMs down to a few degrees can result in a significant improvement of the energy resolution.

In any gamma camera equipped with SiPMs the number of pixels used to collect the charge should be optimized to achieve the best possible energy resolution. In practice, this implies discarding all those pixels in which the ratio of collected photons to integrated dark counts is above an acceptable level. That level will depend on the DCR value and also slightly on the pixel size. In our simulated system, we found that, if SiPMs are operated at room temperature, the optimal number of LASiPs to be used to measure the energy should be ∼60 with our smallest pixels, LASiP 1, and ∼15 with our largest ones, LASiP 4. In both cases, the total number of SiPMs of 6×6 ${\mathrm{mm}}^{2}$ involved is ∼600, which is only ∼15% of the ∼4000 SiPMs that would be needed to cover the whole area of the scintillator.

As expected, pixel size does have a significant impact on the spatial resolution. We have also shown that the way in which the LASiPs are distributed is also very relevant. Most large-area SiPM pixels (often referred as ‘tiles’) have a square geometry (e.g., [27]), which is probably the most natural and easiest way of building a SiPM matrix. Our study suggests that this geometry would not be the most efficient for a gamma camera. Using our reconstruction algorithm, the pixels with a honeycomb geometry provided a much better spatial resolution than those with a square one. For instance, with LASiP 8 we could achieve a spatial resolution of 2.2±0.2 mm at room temperature, which can be compared with the 3.5±0.4 mm we achieved with LASiP 3, which has almost the same size. Even when we group the SiPMs in the ’unnatural’ way of LASiP 5, the overall spatial resolution is much better than when the same SiPMs are grouped in square pixels. The image reconstruction with LASiP 5 is more sensitive to the position of the source and hence a honeycomb geometry like the one of LASiP 8 should be preferred. The spatial resolution is also degraded by SiPM noise and this effect can also be mitigated by cooling down the SiPMs, although including a cooling system for thousands of SiPMs in a small volume might be challenging.

A standard full-body SPECT camera has a typical intrinsic spatial resolution of ∼5 mm [26]. Even if, as we mentioned before, this should not be directly compared to the values we obtained through simulations, the results we found are encouraging and suggest that cameras based on large LASiPs could be competitive. Moreover, since the spatial resolution we found with LASiP 8 is relatively low, it might be possible to build even larger LASiPs and still achieve a reasonable performance, especially when in most SPECT scans the actual resolution will be dominated by the resolution of the collimator.

Since this was a simulation study focused on optimizing the LASiP design, we did not discuss other potential challenges that could be associated to the actual implementation of the proposed solution in a real camera. One challenge would be dealing with the heat that could be generated in a camera full of sensors enclosed in a thick lead shielding, especially if their readout electronics are also placed inside. Indeed, if power-consuming electronics (e.g., ADCs) had to be held close to the SiPMs, heat dissipation would become a real issue. Fortunately, since SPECT does not have strong requirements in terms of time resolution, probably most of the electronics could be placed outside the camera, having only the SiPM decoupling circuit inside. Moreover, several channels could be readout and digitized using low-power ASICs [28,29]. Indeed, with the development of these kind of ASICs capable of digitizing several tens of channels with a few mW, it may become easier to manage more channels in the same camera.

In this work we used a MLE techniques for the image reconstruction, which are known to provide much better spatial resolution than centroid methods. Other algorithms based on Machine Learning techniques could probably provide comparable or better results (see [30] for a recent review). However, it should be noted that both MLE and Machine Learning techniques require higher computing resources and longer computations times. This could be an issue when processing real data at the clinical level, as the need for higher computing resources would raise the cost of the system and the results of the scan might need longer processing times.

A full-body SPECT camera based on LASiPs could be comparable in performance (or better) to a standard one based on PMTs but would provide several advantages. What we believe would be the main one is the reduction of the size and weight of the camera. With smaller and lighter cameras, the cost and the complexity of the whole SPECT scanner could be reduced. Additionally, the lifetime of SiPMs is much larger since they do not suffer from aging and they would simplify the power management as they do not require high voltage. A camera based on SiPMs could never achieve the energy resolution of a CZT one, but it would be much cheaper.

## 5. Conclusions

In this work, we studied, through Monte Carlo simulations, the feasibility of developing large (e.g., full-body) SPECT cameras employing large SiPM pixels. The solution we proposed in [2] to overcome the lack of commercial large-area SiPMs consisted of summing the currents of several SiPMs to build what we called LASiPs. Here, we provided tools that could be useful to optimize the LASiP size and shape when building a large gamma camera.

This study showed the huge impact that LASiP size and noise can have in the performance of a SPECT camera. Dark counts degrade both the energy and spatial resolution of the system. This effect could be largely mitigated by cooling down the SiPMs to a few degrees Celsius. In addition, we found that the geometry of the pixel also has huge impact. Pixels with a honeycomb shape/distribution provide a significantly better spatial resolution than square-shaped pixels. Among the configurations we studied, probably LASiP 8, which is more than 20 times larger than standard commercial SiPMs, provides the best compromise between number of readout channels and performance. Moreover, our results suggest that, if using a honeycomb geometry, there is still margin to build even larger pixels and still achieve a competitive spatial resolution.

## Figures and Tables

**Table 1:** LASiP configurations studied in this work.

Model	Nr of SiPMs per LASiP	Pixel Area [${\mathrm{mm}}^{2}$]	Shape
1	9	324	square
2	16	576	square
3	25	900	square
4	36	1296	square
5	24	864	flower
6	7	252	comb
7	19	684	comb
8	24	864	comb

**Table 2:** Energy resolution obtained with LASiP 1–5 at different noise levels.

	Energy Resolution [%]			
Model	No Noise	Cooled-SiPM Noise	Room-SiPM Noise	Hot-SiPM Noise
1	8.1 ± 0.1	8.4 ± 0.1	9.0 ± 0.1	9.4 ± 0.1
2	8.2 ± 0.1	8.5 ± 0.1	9.2 ± 0.1	9.9 ± 0.1
3	8.2 ± 0.1	8.6 ± 0.1	9.5 ± 0.1	10.3 ± 0.1
4	8.2 ± 0.1	8.7 ± 0.1	9.6 ± 0.1	10.6 ± 0.1
5	8.2 ± 0.1	8.6 ± 0.1	9.6 ± 0.1	10.4 ± 0.1

**Table 3:** Mean intrinsic spatial resolution obtained from reconstructed images of linear sources, using the different LASiP configurations studied.

	Mean Intrinsic Spatial Resolution [mm]	
Model	No Noise	Room-SiPM Noise
1	1.4 ± 0.3	1.6 ± 0.2
2	1.7 ± 0.4	2.0 ± 0.4
3	2.5 ± 0.3	3.5 ± 0.4
4	4.0 ± 1.2	6.0 ± 1.0
5	1.5 ± 0.4	2.1 ± 0.6
6	1.0 ± 0.1	1.2 ± 0.1
7	1.4 ± 0.1	1.7 ± 0.1
8	1.6 ± 0.1	2.2 ± 0.2

**Table 4:** Mean intrinsic spatial resolution obtained with the different LASiP configurations studied.

	Mean Intrinsic Spatial Resolution [mm]			
Model	No Noise	Cooled-SiPM Noise	Room-SiPM Noise	Hot-SiPM Noise
1	1.4 ± 0.3	1.4 ± 0.2	1.6 ± 0.2	1.6 ± 0.1
2	1.7 ± 0.4	1.9 ± 0.3	2.0 ± 0.4	2.2 ± 0.2
3	2.5 ± 0.3	2.6 ± 0.3	3.5 ± 0.4	4.0 ± 0.2
4	4.0 ± 1.2	4.8 ± 1.2	6.0 ± 1.0	8.4 ± 1.5
5	1.5 ± 0.4	1.6 ± 0.4	2.1 ± 0.6	2.5 ± 0.9

## Data Availability

The simulation model can be shared upon reasonable request to the authors.
